# Absence of Spatial Tuning in the Orbitofrontal Cortex

**DOI:** 10.1371/journal.pone.0112750

**Published:** 2014-11-11

**Authors:** Lauren E. Grattan, Paul W. Glimcher

**Affiliations:** 1 Center for Neural Science, New York University, New York, New York, United States of America; University College London, United Kingdom

## Abstract

There is limited data in the literature to explicitly support the notion that neurons in OFC are truly action-independent in their coding. We set out to specifically test the hypothesis that OFC value-related neurons in area 13 m of the monkey do not carry information about the action required to obtain that reward – that activity in this area represents reward values in an abstract and action-independent manner. To accomplish that goal we had two monkeys select and execute saccadic eye movements to 81 locations in the visual field for three different kinds of juice rewards. Our detailed analysis of the response fields indicates that these neurons are insensitive to the amplitude or direction of the saccade required to obtain these rewards. Our data thus validate earlier proposals that neurons of 13 m in the OFC encode subjective value independent of the saccadic action required to obtain that reward.

## Introduction

The orbitofrontal cortex (OFC) plays a prominent role in decision-making research. Most recently, Walker's area 13 m, the most medial section of the OFC, has been described as carrying information about goods in an economic sense rather than information about actions [1]. The area receives projections from a wide range of areas including the gustatory cortex, amygdala, rhinal sulcus, thalamus, ventral tegmental area, premotor areas, along with other prefrontal cortical areas [2], [3]. Early electrophysiological studies noted strong responses in the OFC to gustatory information, such as flavors, presumably due to heavy projections from the gustatory cortex [4].

Lesion studies of the OFC of rhesus macaques have suggested a role for this area in updating reward value representations that are presumed to guide decision-making. A number of studies have shown that monkeys have a difficult time extinguishing previously learned reward values following lesions to OFC [5]–[7]. Recent electrophysiological data supports a role for OFC in value and decision-making. These studies have suggested that individual neurons encode the magnitude of specific rewards. Padoa-Schioppa and colleagues (2008) [8] have shown that different neurons in the OFC respond to different flavors of juice reward. But they also showed that the firing rates of these neurons encode the magnitude or desirability of rewards in a more abstract sense.

Studies like these have given rise to the notion that neurons in the OFC encode the properties of rewards in their firing rates but that these firing rates are *independent of the actions required to obtain those rewards* [9]. The critical idea presented in these papers is that neurons are highly selective for reward-type but completely unselective for the actions that yield those rewards. This conclusion has led to the generation of new models that rest on the idea that decision-making both takes place in Walker's area 13 m of the OFC (amongst other places) and that this process of decision-making is action-independent.

Despite these theoretical steps, there is limited data in the literature to explicitly support the notion that neurons in OFC are truly action-independent in their coding. In fact, those who conduct research in rats suggest significant spatial selectivity of OFC neurons in areas some believe to be homologous to area 13 m [10], [11]. Literature from monkey electrophysiology has also not yet come to a clear consensus on the spatial selectivity of OFC neurons [11]–[16]. Coarse testing of neurons in areas 13 m and 14 by one group has suggested that close to 30% of neurons show lateralization of responses (a preference for left or right movements) [12]. In contrast, other labs using similar approaches have seen little evidence for spatial tuning [13], [16].

Here we set out to test the hypothesis that OFC value-related neurons specifically in area 13 m do not carry information about the action required to obtain that reward – that activity in this area represents reward values in an abstract and action-independent manner. To accomplish that goal we had monkeys select and execute saccadic eye movements to 81 locations in the visual field for three different kinds of juice rewards. These data allowed us to construct detailed response fields for these neurons during a rewarded visuomotor task. As in previous studies, we found that 13 m neurons were highly selective for reward type. Our detailed analysis of the response fields indicates that these neurons are insensitive to the amplitude or direction of the saccade required to obtain these rewards. Our data thus appear to validate earlier proposals that neurons of the OFC in Walker's area 13 m encode subjective value independent of the saccadic action required to obtain that reward.

## Materials and Methods

### Ethics Statement

All animal procedures were developed in association with the New York University Veterinarian, and were approved by the New York University Institutional Animal Care and Use Committee. Monkey subjects were cared for by a team of veterinarians and veterinary technicians including an enrichment specialist who provided daily toys and treats for the animals along with periodic entertainment in the form of a radio or cartoon programming. Analgesia and anesthesia were always employed before any procedure that might induce more than momentary pain or distress. The subjects were pair-housed and received ad libitum food. Subjects were motivated by a fluid regulation design. Following the establishment of a baseline level, animals typically received between 75–85% of their determined daily fluid allotment in the context of successfully performing assigned tasks. The subjects received the rest of their daily allotment in their home cages. These procedures were designed in conductance and in compliance with the *Public Health Service's Guide for the Care and Use of Animals*.

### Subjects

Experiments were performed on two juvenile-adult male rhesus monkeys (*Macaca mulatta*, Subject 1, 13.5 kg, and Subject 2, 7.0 kg).

### Surgical and training procedures

In a sterile surgical procedure performed under isoflurane inhalant anesthesia, a head-restraint prosthesis and scleral search coil were implanted using standard techniques described in detail elsewhere [17]. After surgery, animals received analgesics for a minimum of 3 days. Antibiotic prophylaxis was initiated intra-operatively and continued for a minimum of 3 days.

After a 6-week recovery period that facilitated the osteo-integration of the implanted bone screws, access to water was restricted, animals were habituated to head restraint, and then trained to perform oculomotor tasks for a fruit juice or water reward.

During data collection, horizontal and vertical eye position signals were sampled at 500 Hz using an eye coil system. Tri-state LEDs, which could be illuminated to appear red, green, or yellow to normal human observers, served as visual stimuli. LEDs were fixed on a tangent screen placed 139.7 cm (55 inches) from the eyes of the animal. 81 of these LEDs formed a grid of points, separated by 4°, spanning 36° horizontally and 36° vertically which served as the targets of juice-reinforced saccades.

**Figure 1:**
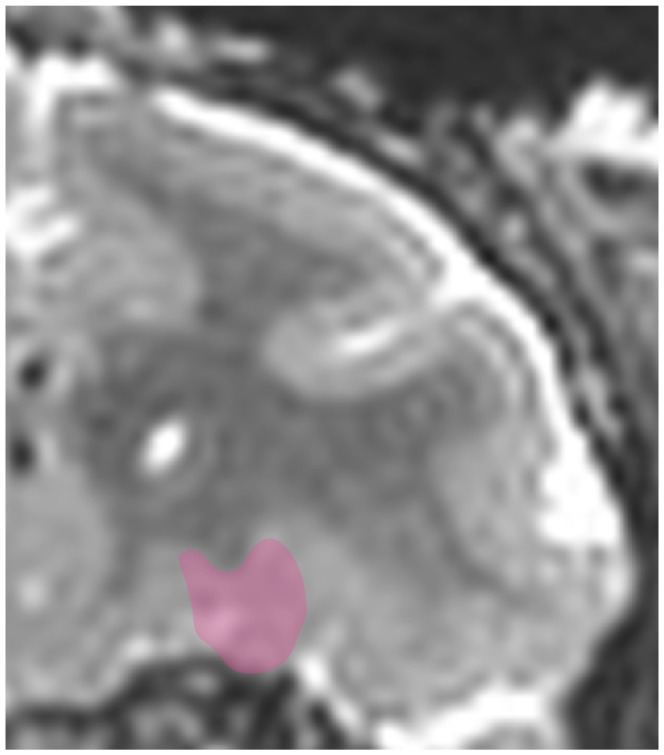
MR Image. Subject 1's MRI image depicts the center of the recording chamber. For reference, a transparent image of a macaque brain atlas reveals the target as Walker's area 13 M.

Following training, a second sterile surgical procedure was performed. Monkeys were implanted with a Cilux recording chamber (Crist Instruments) targeting area 13 m (36 mm caudal and 8 mm lateral to the intersection of the midsaggital and interaural planes) in the left hemisphere for both monkeys. Chambers were implanted using standard surgical techniques described in detail elsewhere [17]. Chamber location was verified using anatomical magnetic resonance imaging (3T; Siemens). Figure 1 illustrates a coronal slice from the MR image representing the center of subject 1's chamber.

### Behavioral Techniques

Animals were trained to associate red, green, or yellow LEDs with 3 different juice-flavored rewards of 0.2 ml. Each trial began with the illumination of central yellow LED which subjects were required to fixate within 700 ms. On 82% of trials a single eccentric, yellow, green, or red LED was illuminated 300 ms after gaze was aligned within 2° of the fixation stimulus. After a further 1000 ms delay, the fixation stimulus was extinguished, cueing the subject to shift gaze to within 2° of the eccentric target and within 350 ms in order to receive the reinforcement associated with the colored light that appeared on the screen. The eccentric target appeared in one of the 81 different locations that made up the 4°×4° target grid which spanned 36°×36° of visual space. On 18% of trials, which we refer to as *choice trials*, two eccentric LEDs were illuminated *simultaneously* representing 1 of 3 different pairs of color LEDs (red and yellow, red and green, or yellow and green), 300 ms after gaze was aligned within 2° of the fixation of stimulus. After a further 1000 ms delay, the fixation stimulus was extinguished, cueing the animal to shift gaze to either of the illuminated targets within 350 ms in order to receive the reinforcement associated with the color of the stimulus he chose.

### Microelectrode Recording Techniques

At the start of each recording session a 23-gauge stainless steel guide tube was positioned in a support grid (1 mm spacing; Crist Instruments) and inserted through the intact dura. A tungsten electrode (8–10 MOhms impedence; FHC) was lowered through the guide tube using a hydraulic micropositioner (KOPF Instruments). Electrophysiological signals were amplified, bandpass filtered, and individual neurons were isolated based on waveform characteristics. Times of spike occurrence were recorded by computer using a 1-µs internal clock. Once a cell was isolated 250–1200 trials were presented in which the location of the eccentric target(s) and the color of the eccentric target varied randomly from trial-to-trial.

### Analysis

The goal of our analysis was three-fold. First, we analyzed the choice behavior of the animals on the two target *choice trials* to determine whether the animals showed clear and consistent preferences with regard to (target color and) juice reward type. We did this to ensure that animals had learned to associate target colors with reward types. An indication of reward preferences through behavior indicated that subjects established ideosyncratic economic values for the rewards. Second, we analyzed the neuronal firing rate data from single target trials to determine whether neuronal firing rates in our dataset clearly discriminated between juice reward-type, as has been repeatedly demonstrated in this area in the past. This served as an internal check that the neurons we examined were similar to those that have been studied previously in this area. To formally test the hypothesis that these neurons are similar to the neurons recorded in Padoa-Schioppa (2006) [14] we measured the reward selectivity of each neuron by averaging firing rates across juice flavors. Third, we analyzed the firing rate data from these single target trials for any evidence that the location of the target, varied systematically over 81 spatial locations, influenced the firing rates of OFC neurons.

To accomplish the second and third goals, we recorded, for each trial, the location of the eccentric visual target and the number of action potentials produced during two intervals 500 ms in length: (1) *Early Analysis*, 400 ms following illumination of the eccentric visual target, and (2) *Late Analysis*, 350 ms following the onset of juice delivery. To choose these analysis periods we considered the analysis periods used by other scientists recording from the OFC (e.g., Padoa-Schioppa and Assad, 2008) because it is their hypothesis that these neurons are spatially untuned during specific trial epochs which we hoped to examine. Thus our epoch selection was driven by existing data, though our precise msec by msec windows were adjusted from those in the literature to align with the timing of our task. This information allowed for the construction of detailed response field plots for each neuron at each of these time points. (We also generated more continuous temporal analyses, which confirmed that our findings were not unique to the temporal epochs we examined.) These response field plots were used to assess the spatial tuning of each neuron during target onset and reward delivery and thus to test the hypothesis that these neurons are not modulated by saccadic-action properties. To test the hypothesis that neurons were insensitive to saccadic-action properties three “models” were fit to the data from each neuron during each interval and the quality of these fits was compared using the Akaike Information Criterion (AIC).

The first model tested the hypothesis that OFC neurons, like neurons in frontal eye fields [18] or area LIP [19] showed a Gaussian tuning with regard to horizontal and vertical saccade amplitude. A two-dimensional Gaussian model was thus fit to the raw data of the response fields measured for each cell as was originally suggested for posterior parietal cortex [20]. The Gaussian model has five free parameters: the horizontal and vertical position of the center, the horizontal and vertical standard deviations (sigmas), and baseline firing rate. The model was constrained so that the center of the Gaussian lay within 36° of the plot origin (the origin is defined as a position straight ahead of the subject) and so that the diameter (standard deviation) of the Gaussian was not larger than the region spanned by our data (36°). Parameters of the model were estimated using a Nedler-Mead simplex iterative fit that minimized the squared Cartesian distance between the Gaussian model and the raw data (Matlab) [21].

To test the simpler alternative hypothesis that, within the limits of our dataset, the neuronal firing rates were modulated as *any* linear function of horizontal and vertical saccade amplitude, we fit the raw data of the response fields with a two-dimensional planar model that had three free parameters: the slope along the y-axis, the slope along the x-axis, and the baseline firing rate. The parameters of this model were estimated using a least-squares linear regression that minimizes the squared Cartesian distance between the planar model and the raw data (Matlab).

To test the even simpler alternative hypothesis that neurons responded more strongly before or after movements to one hemifield than to the other, a third model was fit to the data. The hemifield model had 2 parameters, *firing rate ipsilaterally* and *firing rate contralaterally*. We omitted data from the vertical midline positions, (0,16), (0,8), (0,4), (0,0), (0,4), (0,8), and (0,16) for this model, and averaged the firing rate across the remaining 37 target positions in each hemifield (right and left). Therefore the hemifield “model” contained two parameters simply representing the overall average firing rate of the neurons whenever the subjects looked at a target in either the ipsilateral or contralateral visual hemifield.

To determine whether any of these three models was a better fit to the data we collected than was a model that included *no spatial information whatsoever*, we compared each of these models to a calculation of the mean firing rate of the neuron during all movements that yielded a particular juice reward. For the purposes of comparing the hemifield model to the “mean” model, the “mean” model excluded the central targets from the overall average and represented firing rates in response to 74 stimulus locations, rather than 81. To objectively assess the best model for describing the data from each neuron we used the Akaike Information Criterion (AIC), which estimates the information lost by approximating the true process underlying the data by a particular model [22]. For each model the AIC is computed as
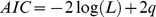
Where L is maximized log-likelihood of the model-fit and q is the number of parameters. AIC allows for a description of the interplay between likelihood estimates and the number of parameters used; likelihood estimates decrease as more parameters are used to describe the data. AIC allows one to ask the question of at what point do the additional parameters stop providing more information and guards against an over-fit. We compared our three model AIC values to the other models as well as to an AIC value that would encapsulate no spatial tuning.

## Results

Eighty-four OFC neurons (Subject 1, n = 48; Subject 2, n = 36) from area 13 m were studied while subjects completed a minimum of 250 reinforced, delayed saccade trials and 30 free-choice trials. We collected data from the average neuron for 520 trials (s.d. 158).

### Behavior During Choice Trials

**Figure 2:**
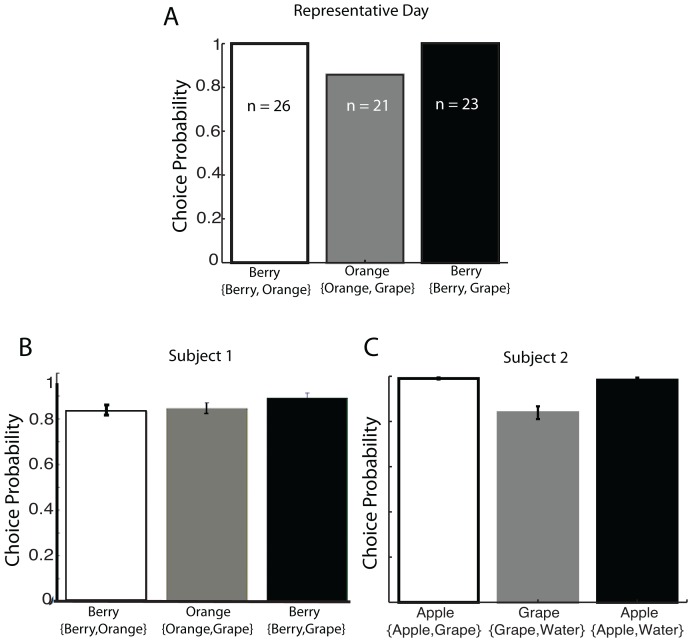
Transitive Behavior. (A) Representative day of choices made during free choice trials: Subject 1 performed a total of 70 free choice trials during one recording session. Plotted are histograms representing the probability the subject chose the preferred juice flavor (berry or orange) given the juice pairing ({berry, orange},{orange, grape},{berry, grape}). During the representative session, the subject chose berry juice over orange juice consistently (n = 26), orange of grape juice with a probability of 0.83 (n = 21), and berry juice over grape just consistently (n = 23). During this session the subject exhibited transitive behavior with berry**≥**orange**≥**grape. (B) Across the 48 recording sessions used in the neural analysis for Subject 1. The subject's preferences remained consistent. The subject chose berry juice over orange juice (m = 0.8405, se = 0.0265), orange juice over grape juice (m = 0.8504, se = 0.0287), and berry juice over grape juice (m = 0.899, se = 0.0201). The monkey's behavior exhibits robust transitivity and consistent preferences indicating that the subject assigned value to the stimuli representative of the three juice flavors. (C) Analysis across the 36 recording sessions used in the neural analysis of Subject 2. The subject's preferences remained consistent. The subject chose apple juice over grape juice (m = 0.99 s.e. = 0.0042), grape juice over water (m = 0.8387, se = 0.0282), and apple juice over water (m = 0.9830, se = 0.0110). The monkey's behavior exhibits robust transitivity indicating that he has learned the associative value of the stimuli.

In order to ensure that subjects understood the relationship between target stimulus color and the type of juice it provided, subjects were repeatedly asked to choose between pairs of saccadic targets. We looked for consistency and transitivity in their choices as evidence that they could discriminate effectively between the stimuli, and the rewards they offered. Subjects were thus asked to choose between two of the three juice flavors on each free-choice trial each recording day. As there were three possible colors/flavor comparisons, the three pairwise comparison trials were presented a minimum of 10 times each during each recording session. Figure 2a plots the choice behavior of a single subject from a single representative day; the subject always (100%) chose berry flavor over orange (n = 26), chose orange over grape flavor at probability 0.85 (n = 21), and always (100%) chose berry over grape flavor (n = 23). The subject exhibits highly transitive behavior on the representative day preferring Berry to Orange and Orange to Grape. Figure 2b and c aggregate all choice data from each subject. Across 48 recordings, Subject 1 chose berry flavor over orange (m = 0.8495, se = 0.0265), orange flavor over grape flavor (m = 0.8504, se = 0.0287), and berry flavor over grape flavor (m = 0.8990, se = 0.0201). Subject 2 exhibited similarly consistent preferences across 36 sessions by choosing apple flavor over grape flavor (m = 0.99, se = 0.0042), grape flavor over water (m = 0.8387, se = 0.0282), and apple flavor over water (m = 0.9830, se = 0.0110). The consistent and transitive behavior suggests that the subjects understood that the stimuli have flavors associated with them and that the subjects assigned stable preferences amongst those stimuli/flavors.

### Neural Activity as a Function of Reward Type

**Figure 3:**
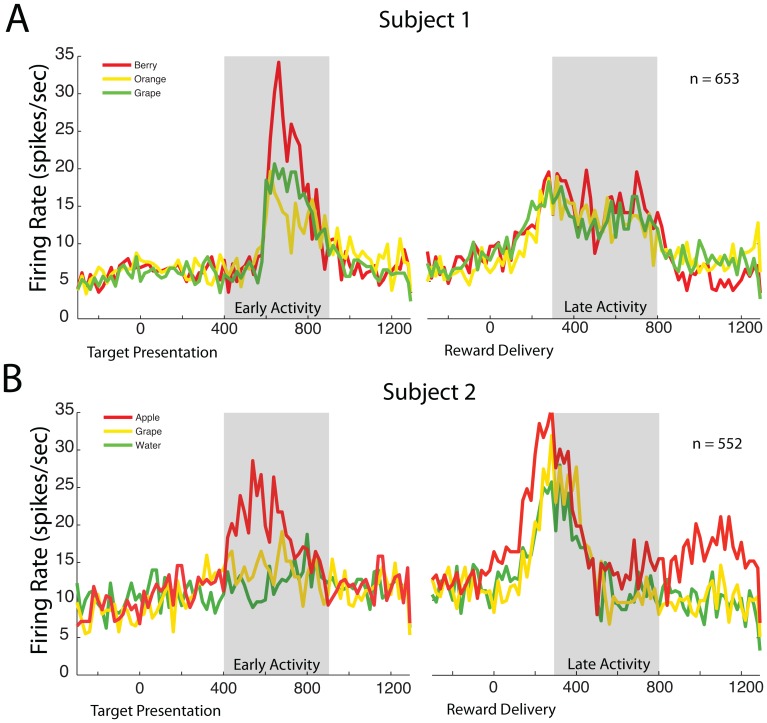
Example neurons from each subject. Each graph plots spikes per second as a function of time in milliseconds and each line represents the average spike rate of the neuron when the subject was presented with one of three rewards. (A) For this particularly neuron the firing rates following stimulus presentation are statistically different (ANOVA, F(2,652) = 3.63, p = 0.0271), while the firing rates following the receipt of reward are not statistically different (ANOVA, F(2,652) = 1.67, p = 0.1889). (B) The example neuron for Subject 2 has statistically different responses to the presentation of the reward stimuli (ANOVA, F(2,551) = 4.11, p = 0.017) and the neurons responds statistically differently to the rewards (ANOVA, (2,551) = 10.96, p<0.001.

We collapsed the data across all stimulus locations and separated the data by firing rates as a function of the reward type. We recorded neurons from the same location as Padoa-Schioppa (2006). To ensure that those neurons were functionally similar, we tested their selectivity for juice flavor. By using a 100 ms sliding window across the duration of a trial and a repeated measures ANOVA with a cut-off of p = 0.05, we found 65% of our neurons from Subject 1 were significantly selective for reward type during the trial and 54% of the neurons from Subject 2 were significantly selective for reward type during the trial. Figure 3 plots two example OFC neurons (one from each of the two subjects) collapsed across all spatial locations (Fig. 3a: n = 653 trials Fig. 3b: n = 552). In figure 3a, the red line plots average firing rate as a function of time for all trials rewarded with berry juice, green represents trials rewarded with grape juice, and yellow represents trials rewarded with orange juice. The neuron responds differently to the different reward types during the target period (ANOVA, F(2,652) = 3.63, p = 0.0271), but does not have statistically different responses to the three reward types in the period following reward delivery (ANOVA, F(2,652) = 1.67, p = 0.1889). In figure 3b, red represents grape juice, yellow represents apple juice, and green represents water. Subject 2's example neuron responds differently to the three reward types following stimulus presentation (ANOVA, F(2,551) = 4.11, p = 0.0170) and following reward delivery (ANOVA (2,551) = 10.96, p<0.001). The firing rates for the example neuron from Subject 2 correspond with Subject 2's preferences. However, the firing rate for the example neuron from Subject 1 does not correspond with the subject's preferences. Padoa-Schioppa (2006) showed that neurons in the OFC are capable of encoding a complex representation of value by representing three different signals: *offer value* (value of one of the juices offered), *chosen value* (the value chosen by the subject in any given trial), and *taste* (a binary variable identifying the chosen juice). We did not record enough free choice data from the subjects to be able to measure the responses of the neurons while the subjects made a choice, and therefore, did not expect to always observe firing rates corresponding either with the subjects' preferences or with other choice-based studies made in this brain area. But most importantly, our data show that more than half of the neurons we encountered, like many of those in the literature [8], [23], show a clear response to reward.

### Neuronal Response Field as a Measure of Spatial Structure

**Figure 4:**
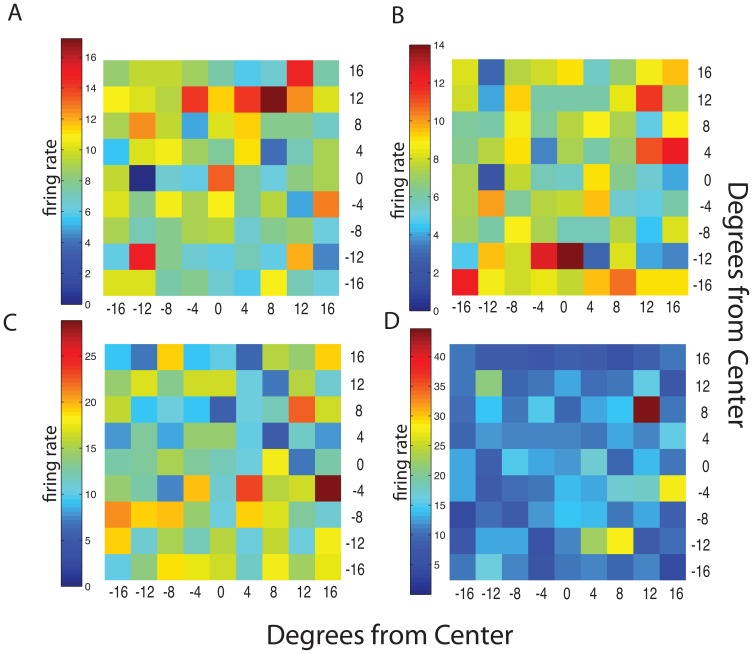
Spatial Grid. Each square represents the average firing rate of the neuron at the given location in the Subject's visual field collapsed across the three juice flavors. (A) and (C) represent firing rates for Subject 1 and 2, respectively, during the early activity following stimulus presentation and (B) and (D) represent firing rates for Subject 1 and 2, respectively, during the late activity following reward delivery.

In order to determine whether there was any spatial structure in the response fields we measured, we began by aggregating data from each neuron across juice types. We then plotted neuronal activity during the 400 ms after target onset (early activity, indicated by the first grey bars in Fig. 3) and the 350 ms following juice delivery (late activity, second grey bars) against the horizontal and vertical position of the targets, in 4° bins. Figures 4a and b plot these response fields for the early and late activity, respectively. The plots in Figure 3a and Figures 4c and d plot data for the early and late activity, respectively, from the example neuron shown in 3b. Without any statistical analysis, it seems clear that these neurons do not show any discernible spatial selectivity.

### Model Fits and Quantifying Spatial Selectivity

To search systematically for evidence of spatial tuning that may be too subtle to detect intuitively, throughout our database we examined the goodness of fit of three response field models: A 2-dimensional gaussian, a 2-dimensional linear (or planar) response field and a simple hemifield-model, which differentiates rates for movements only with regard to the left and right hemifield. The goodness of fit of these models was compared to the hypothesis that neurons have a fixed mean rate that does not vary with movement or stimulus properties. (See methods for details.)

**Figure 5:**
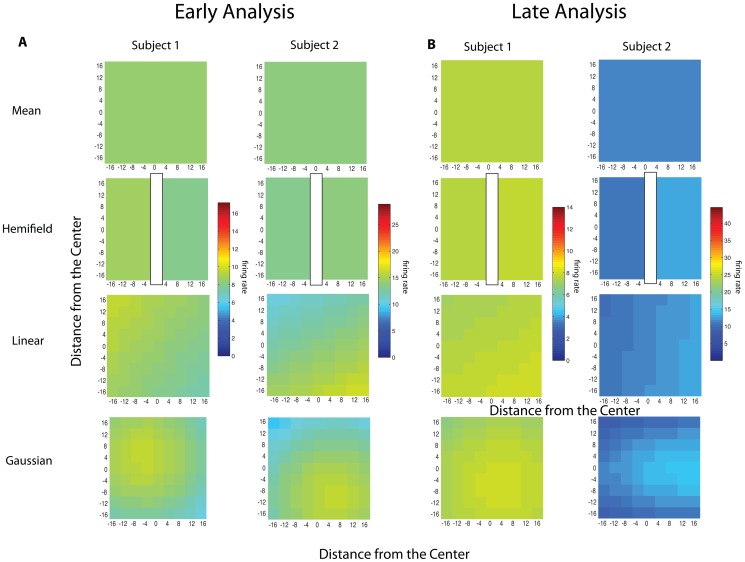
Model Fits to Data. Using the scale from figure 4, which scales by the average firing rate for each stimulus location, these are heat maps across the visual field of the model fits for example neurons from subject 1 and subject 2 during the (A) early period of analysis and the (B) late period of analysis.

**Figure 6:**
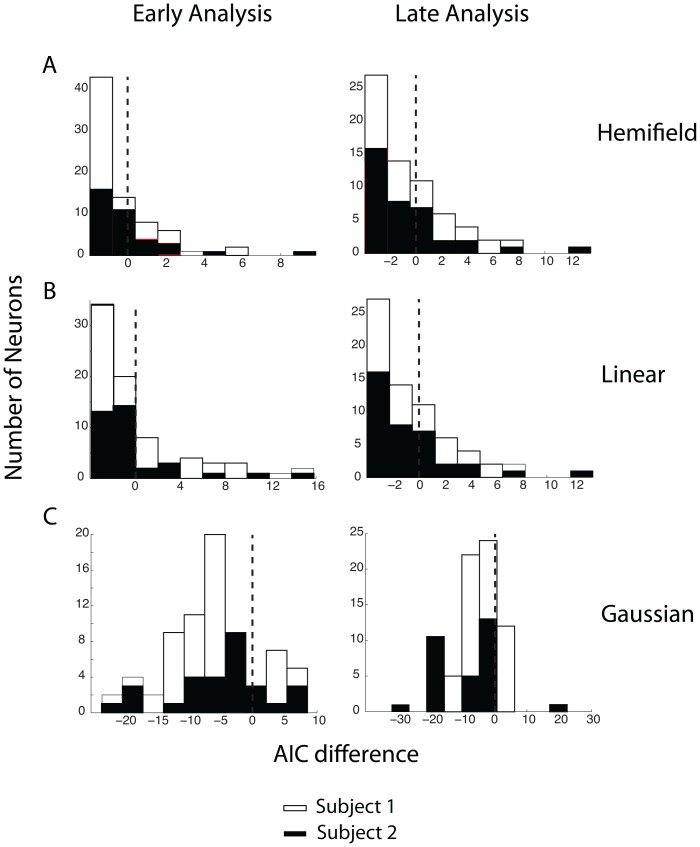
AIC Differences. Histograms of the difference in the AIC; white represents Subject 1 and black represents Subject 2 during the early analysis and late analysis. The lower axis is constructed by taking the AIC number for a uniform firing rate and subtracting the AIC number for the (A) hemifield, (B) linear, and (C) gaussian models. Neurons to the left of zero are better fit by the ‘mean’ model. For Subject 1, the binomial probabilities of observing so many negative AIC differences if the models fit equally well are all less that 0.05 (early analysis hemifield: p = 0.0336, late analysis hemifield: p = 0.006, early analysis linear: p = 0.0014, late analysis linear: p = 0.0349, early analysis gaussian: p = 0.0001, late analysis gaussian: p = 0.0001). For Subject 2, the binomial probabilities of observing so many negative AIC differences by chance are at or below 0.055 (early analysis hemifield: p<0.0001 late analysis hemifield: p<0.00005 early analysis linear: p = 0.055, late analysis linear: p = 0.055 early analysis gaussian: p<0.00005, late analysis gaussian: p = 0.0087).

To give an example of a typical set of fits, Figure 5 plots best fitting models to the raw data in Figures 4a and b. The mean represents a model that describes the response field as firing rates randomly distributed around a mean. The hemifield model represents an ipsilateral or contralateral bias. The linear model encodes changes in firing rate as a linear function of position. Finally, the 2-dimensional gaussian plots firing rate as a gaussian function of target position. Note in figure 5 how obviously similar all model fits are to the mean ‘model’ shown at the top for this neuron. Figure 6 plots the quality of each of these fits using the Akaike Information Criteria (see methods). As is obvious, the AIC reveals that the two example neurons do not show evidence of spatial tuning as the AIC value of the ‘mean model’ is negligible or lower than the AIC values for the other models (Subject 1, Early Analysis: linear AIC difference = −3.83, gaussian AIC difference = −10.8, hemifield AIC difference = −1.20E+00; Late Analysis: linear AIC difference = −2.85, gaussian AIC difference = −3.05, hemifield AIC difference = −2.00; Subject 2, Early Analysis: linear AIC difference = −6.8156, gaussian AIC difference = −1.0140, hemifield AIC difference = −7.2006; Late Analysis: linear AIC difference = −1.5156, gaussian AIC difference = 0.0278, hemifield AIC difference = −4.9700). A uniform firing rate, encoding only features of the reward but not of the action required to obtain that reward, accounts for the data better than any of the spatially structured models.

To quantify our entire database, we performed these same 8 fits for each neuron and derived a measure of the AIC for each fit for each neuron. We then compared each pair of fits (mean versus hemifield, mean versus linear, and mean versus gaussian) by simply subtracting the AIC measures. Figure 6 plots these comparisons during the early and late intervals. Observations to the left of the dotted line indicate neurons for which the mean firing rate alone accounts for the neuronal response better than any of the models. The plots show that while most of the AIC values for the individual neurons lie to the left of zero. Thus, the neurons are overall best represented by a uniform firing rate. There are, however, some neurons that have firing rates better described by one of the other model fits. To determine the statistical significance of encountering these occasional AIC values supporting one of the other models in our neuronal population, we calculated the binomial probability of observing this distribution of AIC differences, given the assumption that all 4 ‘models’ were equally good fits to the data. We found that the binomial probability that this distribution of AIC values would be observed for any model comparisons against the mean (uniform distribution of firing rates) were always less than 0.05 for Subject 1 (early analysis hemifield: p = 0.0336, late analysis hemifield: p = 0.006, early analysis linear: p = 0.0014, late analysis linear: p = 0.0349, early analysis gaussian: p = 0.0001, late analysis gaussian: p = 0.0001) and at or below a probability of 0.055 for Subject 2 (early analysis hemifield: p<0.0001 late analysis hemifield: p<0.00005 early analysis linear: p = 0.055, late analysis linear: p = 0.055 early analysis gaussian: p<0.00005, late analysis gaussian: p = 0.0087).

**Figure 7:**
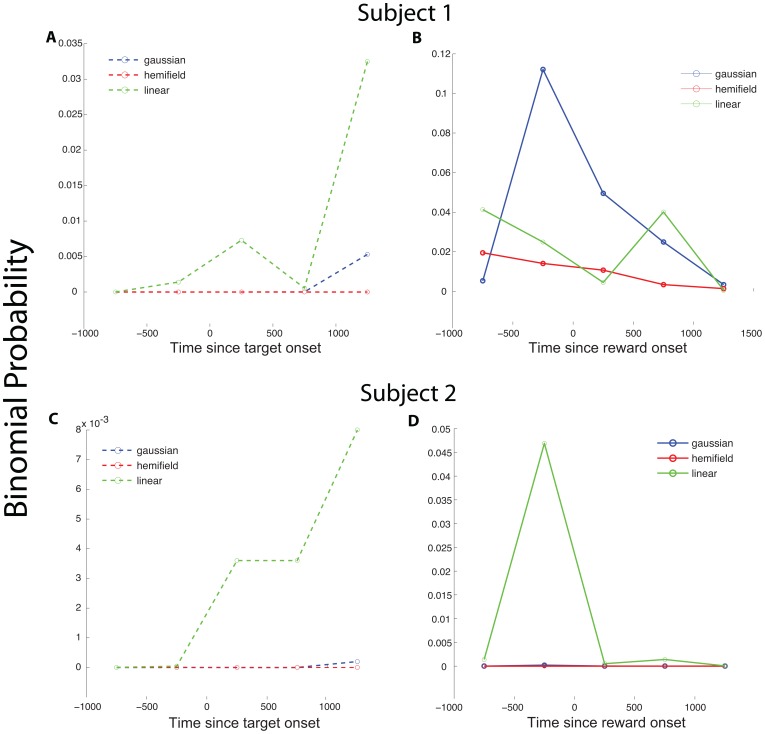
Binomial probabilities 7 time points across the trial. Binomial probabilities are calculated as the probability (p = 0.5) of finding k = number of negative AIC differences given the total number of neurons (n) for Subject 1 (a and b) and Subject 2 (c and d). The sliding windows are aligned to the target onset (a and c) and the reward onset (b and d). On each of the graphs the blue, red, and green lines represent the binomial probabilities for the difference between the gaussian, hemifield, and linear models, respectively, and the mean.

Finally, we wanted to eliminate the possibility that our findings were specific to the two specific analysis periods we selected based on the timing of previous experiments. We therefore broke up each trial into sequential non-overlapping 500 ms windows spanning the entire trial. We then calculated the AIC values for all of the fits in all of these windows (figure 7). For each epoch we therefore computed the binomial probability that the number of negative AIC values out of the total number of neurons per subject was that which would be expected by chance. We found this additional analysis largely confirmed our findings. The binomial probability that the ratio of positive and negative AIC values occurred by chance is below 0.05 for all time periods and all model comparisons, except for one. In that case, which was an epoch of time during which saccades were produced, the Gaussian model weakly outperformed the mean as a firing rate descriptor. While there have been only few detailed numerical studies of activity in this area during saccade generation, and while it should be noted that firing rates are low during this period, this finding may suggest some degree of spatial selectivity under these limited conditions.

**Figure 8:**
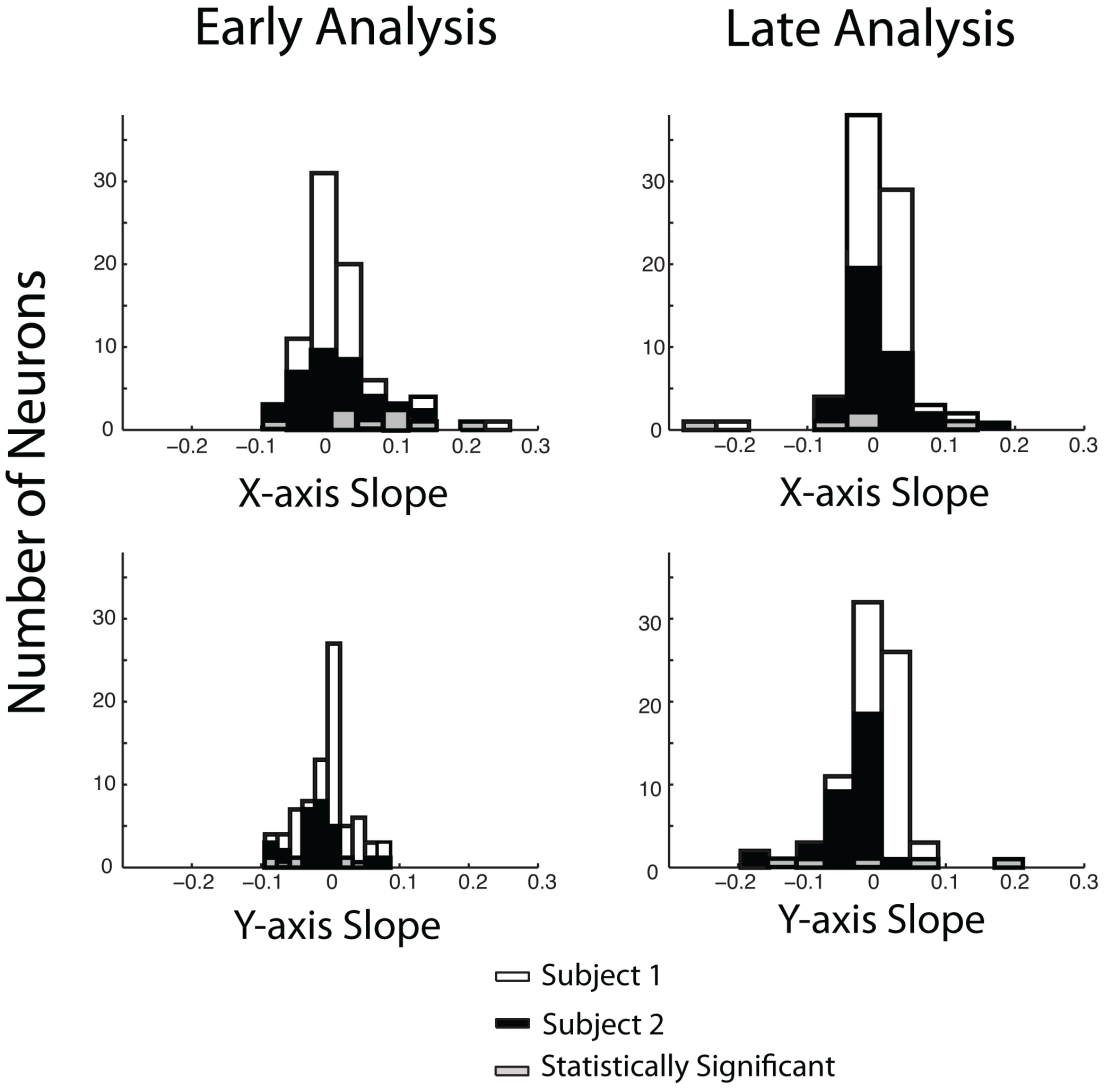
Plane Fitted Slopes. Histogram plots of the slopes of the plane along the x and y axis during the early and late analysis using the linear model. White bars represent Subject 1 and black bars represent Subject 2 with grey representing the linear fits that create a plane statistically different from zero. The statistically significant linear fits do not support the conclusions that there is separate group of neurons for the early analysis (**c** ^2^(1,84) = 55.05, p<0.001) or the late analysis (**c** ^2^(1,84) = 65.19, p<0.001).

To summarize then, all of the histograms, except one, show that the neuronal population does not appear to show any systematic spatial tuning, despite the fact that the majority of these neurons show clear tuning for reward type. However, because the mean and the linear model were very similar and the binomial probability of obtaining negative AIC values in our population exceeded the traditional alpha value of 0.05 for statistical significance in subject two, we performed an additional analysis to compare the mean and linear models. We constructed 4 histograms plotting slopes in the x and y direction during the early analysis and during the late analysis (Figure 8). Note that despite the fact that these models were not much worse than the mean rate “model”, we found fitted slopes were *very close to 0*, and, in fact, only 8 neurons had slopes that were significantly different from zero during the early analysis and only 5 neurons had slopes that were significantly different from zeros during the late analysis. Following this line of reasoning we also note that a chi-square test of significance rejects the null hypothesis that there are two populations of neurons equally dispersed between significantly different from zero and not significantly different from zero during the early analysis (**c** ^2^(1,84) = 55.05, p<0.001) and late analysis (**c** ^2^(1,84) = 65.19, p<0.001).

## Discussion

We tested the hypothesis that orbitofrontal cortex neurons from Walker's area 13 m do not carry information about the saccadic eye movement required to obtain reward. To do this, we created a detailed response field “map” measuring the firing rates of neurons drawn from Walker's area 13 m as a function of stimulus location (and thus saccade metrics) across 81 positions in the visual field. We fit these maps with traditional models of spatial tuning from other domains in neuroscience. Our findings suggests that, for the class of task we examined, the population of OFC value-related neurons we examined in area 13 m *do not carry information about the action required to obtain that reward*; that activity in this population represents reward values in an abstract and action-independent manner. While this is a negative result, these are the most detailed spatial maps of the responses of area 13 m neurons yet gathered in a saccadic task and they seem to confirm the findings by some others that these neurons do not carry spatial information [9], [13], [14], [16], [24]. It might well be argued that our negative result does not mean that these neurons do not carry spatial information about other classes of movement or in other classes of task. This is an excellent point, which must be borne in mind with any negative result. We do note, however, that the methods we used to search for spatial tuning are standard, other studies using similar techniques and similar numbers of trials to study units in other brain areas have found neurons that respond to spatial information in a wide variety of those other areas [25]–[27], therefore we believe that the power in our experiment is sufficient for substantiating our core claim.

In the experiment, the subjects were trained to associate juice flavors to a stimulus color. In figure 2, we show that the subjects develop a preference for stimuli and assume that the preference and value representations are dependent on the juice flavors rather than the colors of the visual stimuli signaling those rewards. We are inclined to this attribution both due to the strong gustatory cortex connectivity to OFC [2] and as a result of previous experiments identifying strong responses in OFC to gustatory information rather than visuo-spectral information [4]. But it is important to note that an alternative explanation for our findings is that a subset of the neurons may show selectivity for the color of the saccadic target and not for the juice flavor or reward quality. While we see the existing data as strong suggesting that this selectivity is for properties of the reward rather than the visual stimulus and support Padoa-Schioppa and Cai (2011) findings that area 13 m encodes value in the absence of action-related information, our data alone cannot resolve this issue.

Our experiment indicates that under basic conditions in an oculomotor task, orbitofrontal cortex neurons do not have any spatially tuned organization, although we did find evidence for value and reward coding like that previous reported in this area [14], [15]. Of late, the hypothesis that prefrontal neurons are capable of coding value in the absence of both choice and action has come under fire. FMRI experiments in humans provide evidence that prefrontal areas are capable of encoding value for a stimulus before action planning or stimulus onset [28]. Additionally, it appears that similar prefrontal areas in humans are capable of encoding the value of a stimulus even in the absence of a decision [29]. In single unit recordings from monkeys there is also evidence that many frontal cortical neurons encode value in the absence of action planning. Padoa-Schioppa (2007) for example recorded neurons from area 13 m and described the neurons as encoding the value of goods in an action-independent framework. Our data support those conclusions.

Our results may be slightly controversial because some groups have found preferential tuning to stimulus location in some parts of the OFC in macaques. For example, Tsujimoto et al. (2009) reported that up to 28% of their neurons encoded a location preference at the spatial resolution of a visual hemisphere (preferring left or right). Wallis and Miller (2003) and Kennerley and Wallis (2009) report that during certain periods within a trial up to 8% and 12%, respectively, of their OFC neurons showed some location selectivity. With regard to this later finding, it should be noted that when comparing our “hemifield model” to a global mean using the AIC, during either the neuronal activity directly following the stimulus presentation or directly following reward delivery 10% of our neurons were modeled better by hemifield selectivity than by a uniform firing (this is also true for a selective analysis of juice responsive neurons). A chi-square test of significance of that finding, the hypothesis that there are two (bimodally distributed) groups of neurons one of which is better represented by a hemifield model and one of which is better represented by the mean model, cannot be statistically supported by the 10% of the neurons observed to be better fit by the hemifield model in our dataset (**c** ^2^(1,84) = 55.05, p<0.001).

The differences in recording location and task design between our experiment and others, however, may also in part explain the apparent inconsistencies between our results and other findings in the literature. We, like Padoa-Schioppa and Assad (2008), restricted our recording location to 13 m specifically to test whether this area carries information about actions or goods. Others have recorded neurons from areas 14 and 13 [16] or from areas 11,12, and 13 [12]. Based on our results, one might well hypothesize that areas 11 and 12 are responsible for the nearly 30% of neurons showing spatial selectivity identified in Tsujimoto and colleagues' experiment. It is also worth noting that both of the above experiments placed a substantial cognitive load on the subject during difficult tasks. It is possible that by enlisting a difficult task, multiple frontal cortical regions are recruited slightly altering the signal out of OFC neurons. Designing a task that requires monkeys to use significant and long-lasting cognitive load while thoroughly mapping the spatial field may elucidate the differences in tuning properties that we have found in our experiment compared to others.

In any case, our data support the hypothesis that OFC neurons in area 13 m do not carry information about actions and that the population encodes abstract and action-independent reward values. Despite the existence of a few neurons that tested weakly positive for location selectivity, we found no evidence for a separate *population* of neurons that meaningfully encode location selectivity. These data, the most densely sampled spatial tuning data ever gathered in OFC 13 m, agree with Padoa-Schioppa and Cai (2011) suggesting that area 13 m is a value-coding region that does not carry action-related information.
